# Large Solitary Pyogenic Liver Abscesses: A Review of Their Management at a Tertiary Care Hospital

**DOI:** 10.7759/cureus.23170

**Published:** 2022-03-15

**Authors:** Divya Prasad, Manzoor Ahmad, Sachin Katyal, Ajay K Thakral, Musharraf Husain, Mohammad Mohsin

**Affiliations:** 1 Department of Surgery, Hamdard Institute of Medical Sciences and Research, New Delhi, IND

**Keywords:** tertiary care hospital, exploratory laparotomy, pigtail catheterization, usg guided aspiration, pyogenic liver abscess

## Abstract

Background and objective

Liver abscesses are one of the common surgical diseases to be treated as an emergency in any tertiary care hospital in India. The formation of abscesses in the liver is still a major problem and associated with significant morbidity in developing countries. We come across all types of liver abscesses, such as amoebic (most common), pyogenic, mixed, and occasionally fungal. There have been several studies on the percutaneous modality of treatment for pyogenic liver abscesses. Most of the studies suggest that percutaneous catheter drainage (PCD) offers a better approach than aspirations for treating pyogenic liver abscesses. However, a few recent studies suggest that percutaneous aspiration leads to equally good results when compared to percutaneous drainage. In this study, we aimed to review the management of solitary large pyogenic liver abscesses and to assess the effectiveness of ultrasonography (USG)-guided aspiration in the procedure.

Methods

A retrospective study was carried out at the Department of General Surgery of our institute. In this study, a total of 27 patients treated for solitary pyogenic liver abscess were included. All patients with a large liver abscess greater than 5 cm without the features of frank peritonitis were included. These patients were followed up regularly for six months.

Results

The single-attempt USG-guided aspiration was successful in 70.3% of patients. Repeat USG-guided aspiration was performed in 18.5% of patients. In 7.4% of patients, a USG-guided percutaneous pigtail catheter was placed. And only 3.7% of cases required exploratory laparotomy.

Conclusion

Based on our findings, USG-guided aspiration is a fairly efficient method for treating a large solitary pyogenic abscess with acceptable results, shorter hospital stays, and minimal complications.

## Introduction

Liver abscess is one of the common causes of admission to general surgery departments in India. The formation of abscesses in the liver is still a major medical condition with significant morbidity in developing countries. We encounter all types of liver abscesses in clinical practice, ranging from amoebic (most common) to pyogenic, mixed, and occasionally fungal. In the past, appendicitis was known to be the cause of a pyogenic abscess due to portal vein transmission [1]. Gradually, biliary diseases increasingly started to turn into liver abscesses, either of an obstructive pathology or a malignant etiology [2-7]. Other causes such as bacterial endocarditis, sepsis, intravenous substance abuse, liver trauma, and gastrointestinal endoscopy are also etiologically documented [3,8].

Traditionally, a large liver abscess had been treated by a major laparotomy, which had been associated with significant morbidity, longer hospital stay, and even mortality [9]. However, with the advent of radiological interventions and a good selection of antimicrobial agents, the treatment of liver abscesses has now become more conservative. Currently, the most common method of treating liver abscesses is either ultrasonography (USG)-guided percutaneous needle aspiration (PNA) and/or USG-guided percutaneous catheter drainage (PCD) [10,11]. These minimally invasive methods have significantly reduced the morbidity in such patients. USG-guided drainage is usually recommended for pyogenic liver abscesses while guided aspiration is performed for amoebic liver abscess as indicated [12].

There are several studies in the literature on pyogenic liver abscesses, which have generally shown that PCD is better than aspiration given its higher success rate, shorter time for abscess volume reduction, and faster clinical recovery [13-15]. However, a few studies have suggested that percutaneous aspiration leads to equally good results compared to percutaneous drainage [16,17].

The aim of the study is to review the outcomes and effectiveness of USG-guided aspiration in the management of solitary large pyogenic liver abscesses.

## Materials and methods

A retrospective study was conducted from December 2016 to December 2020 at the Department of General Surgery in a tertiary care center in New Delhi, India. All patients aged more than 12 years with a solitary large (>5 cm in size) pyogenic liver abscess were included. Cases of amoebic liver abscess, small pyogenic liver abscesses (<5 cm in size), multiple liver abscesses, patients with signs of peritonitis at presentation, cases involving follow-ups of shorter than six months, and those lost to follow-up were excluded. During the study period, a total of 45 patients were treated for pyogenic liver abscess, but 18 patients were excluded on the basis of the exclusion criteria. Hence, only 27 patients qualified for the final analysis.

We reviewed the clinical records of the included patients to document the following features:

(A) Demographic characteristics

(B) Clinical features such as abdominal pain, fever, jaundice, nausea, and vomiting

(C) Risk factors like chronic alcohol consumption and biliary diseases

(D) The basis of the pyogenic liver abscess diagnosis

(E) Further management

(F) Indications for abscess aspiration

(G) Hospital stay and its indications

(H) Complications

(I) Follow-up characteristics

The continuous variables were expressed as mean ±standard deviation (SD) and the categorical variables were expressed as proportions, ratio, or percentage. The data from the clinical records were charted, and a descriptive analysis of the above-stated characteristics was performed.

## Results

The demographic and clinical characteristics of the reviewed patients are shown in Table 1. The mean follow-up duration was 19.8 ±8.2 months. Fever and abdominal pain were the predominant symptoms. The most frequent risk factor association was that of chronic alcohol consumption. The diagnosis of the pyogenic liver abscess was confirmed with a USG of the whole abdomen, negative serology for amoebic abscess, and positive bacterial culture report of the abscess aspirate. The indication for USG-guided aspiration was the presence of liquefied contents in the abscess area. The aspirates culture revealed *Escherichia coli (E. coli)* to be the most commonly isolated microorganism. The longest dimension of an abscess was considered the size of that abscess. The size of abscesses ranged from 5 to 12 cm.

**Table 1:** Demographic and clinical characteristics of the reviewed patients SD: standard deviation

Characteristics	Observation
Total number of patients	27
Male:female ratio	4.4:1
Age in years, mean ±SD	45.81 ±11.67 (range: 20-70)
Distribution of symptoms (symptom: percentage of patients)	Fever: 85.1%; abdominal pain: 77.7%; nausea: 66.6%; vomiting: 25.9%; jaundice: 22.2%
Risk factors and percentage of patients	Chronic alcoholism: 48.1%; biliary disease: 22.2%; diabetes mellitus: 9.6%; idiopathic: 11.1%
Causative organism based on pus culture (microorganism: percentage of patients)	*Escherichia coli*: 62.9%; *Klebsiella pneumoniae*: 22.2%; *Streptococcus milleri*: 11.1%; *Staphylococcus aureus*: 3.7%

A total of 24 patients were treated by aspiration alone: 19 (70.3%) patients with a single-attempt USG-guided aspiration and five (18.5%) with multiple aspirations (Table 2). Repeat USG-guided aspirations were performed when subsequent USG revealed a residual collection of more than 5 cm size in any plane. Serial abdominal USGs were performed to ascertain the progression of the disease: first at the time of admission, subsequently on the 10th day, at one month, third month, and lastly at the sixth month of USG-guided aspiration. The USG on the 10th day revealed residual collection in five cases, and at one month in a single case. Repeat aspiration was performed in these five cases. Additionally, in two (7.4%) patients, a USG-guided percutaneous pigtail catheter insertion was necessary due to the non-resolving fever and the recurrent formation of liquefied abscesses. An exploratory laparotomy was performed in one (3.7%) patient, as frank peritonitis developed soon after USG-guided aspiration, which did not respond to conservative treatment even after 24 hours.

Initially, all patients received the broad-spectrum antibiotic piperacillin-tazobactam along with metronidazole, which were subsequently changed to culture-specific antibiotics. Patients were kept on intravenous antibiotics during their hospital stay and were discharged on oral ones whenever feasible. The antibiotics were continued for three to four weeks. Table 2 summarizes the types of intervention among the patients.

**Table 2:** Types of intervention in the reviewed cases USG: ultrasonography

S. number	Intervention	Number of patients	Percentage (%)
1	Single-attempt USG-guided aspiration	19	70.3
2	Repeat USG-guided aspiration	05	18.5
	(A) Second aspiration	04	14.8
	(B) Third aspiration	01	3.7
3	USG-guided percutaneous pigtail catheter insertion	02	7.4
4	Exploratory laparotomy	01	3.7

The average hospital stay among patients treated with aspiration alone was 8.2 days (Table 3). The patients were kept in the hospital until they had a resolution of abdominal pain or fever. The patients were discharged once they became asymptomatic, felt well, and remained afebrile for a minimum of 72 hours, after which they were discharged with the advice to follow up in the general outpatient surgery department. These patients were followed up at intervals of one, three, and six months thereafter. Clinical examination was performed at each follow-up, and those patients with any positive signs of liver abscess were radiologically evaluated through repeat USG. All reviewed patients had recovered by the last follow-up and had no signs of abscess recurrence on the six-month follow-up USG. There were no treatment-related complications during the follow-up period.

**Table 3:** Hospital stay of the reviewed cases

S. number	Procedure	Number of patients	Hospital stay (days)
1	Single aspiration	19	7.5
2	Multiple aspirations	5	11
3	Pigtail drainage	2	17.5
4	Exploratory laparotomy	1	15

## Discussion

Pyogenic liver abscess is a bacterial infection of the liver leading to an encapsulated collection of infected fluid inside liver parenchyma. It is a serious illness associated with nonspecific clinical features in some cases, which can delay the diagnosis, leading to high mortality rates in the past [18,19]. The advent of effective antibiotics and improved imaging techniques have helped in early diagnosis and intervention, thereby lowering mortality and morbidity.

Most of the studies on the pyogenic liver abscess have consistently found that males are affected more than females. Kubovy et al. [20] observed a male-to-female ratio of 4:1 in their study. Other studies have found that liver abscesses are twice as common in men, and the male-to-female ratio in one study was 1.32:1 [16,21]. The findings of our study, which included 27 pyogenic liver abscess patients, are in line with those of the above-mentioned studies. Our patients were predominantly male. However, our study findings differed from those of some studies in terms of the age of the affected patients. Some studies have reported much older patients being affected by pyogenic liver abscess, with the patients reportedly aged over 60 years on average [19,21]. Contrary to the above observations, the average age at presentation in our study was 45.81 ±11.67 years. This finding suggests that patients of a slightly younger age group were affected in our geographical areas.

Patients with pyogenic liver abscesses can present in various ways. They may be walk-in patients in the outpatient department and, at the other extreme, they may be too sick to present to the emergency on their own. In our study, fever was the most common complaint among patients with pyogenic liver abscess, followed by abdominal pain, nausea, vomiting, and jaundice. But we had one patient who presented to the emergency; that case was diagnosed as subcapsular rupture of the liver abscess and USG-guided aspiration was performed for it. However, the very next day, the patient developed generalized peritonitis, necessitating exploratory laparotomy for the same. The clinical presentation in our study was consistent with the study by Marianna et al. and other studies [21,22,23], in which the patients showed similar symptoms in a similar order, although the proportion varies.

There were 13 patients in our cohort with a history of chronic alcoholism. Very few studies have attempted to determine the association of alcoholism with liver abscesses [24,25], and nearly half of the patients in our study were alcoholics. Although our study cannot confirm that alcoholism is an etiology for pyogenic liver abscess, it can nonetheless be either a risk factor or a confounding factor, and this topic needs further investigation.

The next common etiology was biliary-related (22%); there are many studies citing biliary disease as the most common or second most common cause of pyogenic liver abscess [2-5,20]. The biliary causes may vary in nature; it can be cholelithiasis, and it can also take the form of malignancy [26,27]. In our study also, biliary diseases were the second most common risk factor. We had six patients in total, out of which four were cases of chronic calculus cholecystitis, and one patient was a case of choledocholithiasis along with cholelithiasis who underwent endoscopic retrograde cholangiopancreatography (ERCP) and stenting. All the patients finally underwent laparoscopic cholecystectomy. There was one patient with cholangiocarcinoma, who got diagnosed with the same as well as pyogenic liver abscess via contrast-enhanced CT (CECT) of the whole abdomen. USG-guided aspiration was done for the abscess and the patient was referred to the oncology department for the management of cholangiocarcinoma.

The pyogenic liver abscess is mostly polymicrobial [28]. The most common organism we found was *E. coli*, followed by *Klebsiella*, and then *Streptococcus* and *Staphylococcus*. There are many studies that have taken into account the organisms isolated and cultured in pyogenic liver abscesses. Overall, *E. coli* is the most common organism, followed by *Klebsiella* [3,4,26]. However, a study from Europe found that after *E. coli*, the next most common organism is *streptococci* [20].

Previously, the only way to drain liver abscesses was by laparotomy. However, over time, non-invasive methods of draining liver abscesses have become more popular and these have many advantages as well. Since the emergence of these methods, many studies have compared PCA with PCD for the treatment of liver abscesses. Many studies clearly state that PCD outperforms PNA in light of its higher success rate, decreased mean time in achieving clinical improvement, achieving 50% reduction in cavity size, and also reduced hospital stay [13,29]; however, some other studies suggest that PNA is as effective as PCD for smaller abscess cavities, but even they have concluded that PCD is better for cavities that are more than 5 cm in size [16,26]. In contrast with the above studies, a few studies consider both PNA and PCD equally effective and recommend PNA as the first-line intervention. This is attributed to its simplicity, cost-effectiveness, reduced hospital stay, and better results in terms of patient comfort and safety. There is also the added advantage of draining multiloculated liver abscess [17,30,31].

Our study aimed to review the effectiveness of USG-guided aspiration in cases of solitary large pyogenic liver abscess (>5 cm) and we found success with single aspiration in more than 70% of patients, although some required two or three aspirations. Only two patients who did not respond to aspiration underwent pigtail drainage. A patient with subcapsular rupture of liver abscess, who developed peritonitis one day after the aspiration, underwent an exploratory laparotomy. A thorough lavage was given followed by the placement of drains, one in the cavity and the other in the right subhepatic space. The patient had a slightly delayed recovery but was discharged after a total stay of 15 days. Overall, we have achieved good results with an average hospital stay of 8.2 days in patients treated with USG-guided percutaneous aspirations alone. All patients were followed up for a minimum period of six months, and none of them showed relapse or any other complications.

The current study has some limitations. Firstly, the study was retrospective in nature and hence has limited value in understanding the impact of the interventions on the outcomes. Secondly, several factors other than those discussed in the current analysis could have contributed to the disease resolution but were beyond the scope of our study. Third, the sample size was relatively small and a larger volume of cases would probably be helpful to make more reliable recommendations. Lastly, a control group is required to show the superiority of the PCD over other treatment options. Therefore, further large-scale prospective studies are needed to gain deeper insights and establish substantial evidence.

## Conclusions

Pyogenic liver abscess is a common surgical emergency. Many treatment modalities are available to treat large pyogenic liver abscesses. Currently, most of the cases are being treated using pigtail catheter drainage. Our findings suggest that USG-guided aspiration is a fairly good method of treating large solitary pyogenic abscesses and is associated with promising results, shorter hospital stays, and minimal complications. Pyogenic liver abscesses have been observed in alcoholic patients at a fairly high rate, which indicates a potential association between pyogenic liver abscess and alcoholism in our geographical area, which may also be the subject of further research studies.
